# Living Alone or With Others and Depressive Symptoms, and Effect Modification by Residential Social Cohesion Among Older Adults in Japan: The JAGES Longitudinal Study

**DOI:** 10.2188/jea.JE20170065

**Published:** 2018-07-05

**Authors:** Kaori Honjo, Yukako Tani, Masashige Saito, Yuri Sasaki, Katsunori Kondo, Ichiro Kawachi, Naoki Kondo

**Affiliations:** 1 Osaka Medical College, Faculty of Medicine, Osaka, Japan; 2 Osaka University, Graduate School of Medicine, Department of Public Health, Osaka, Japan; 3 Department of Global Health Promotion, Tokyo Medical and Dental University, Tokyo, Japan; 4 Research Fellow of Japan Society for the Promotion of Science, Tokyo, Japan; 5 Nihon Fukushi University, Faculty of Social Welfare, Aichi, Japan; 6 Chiba University, Center for Preventive Medical Sciences, Chiba, Japan; 7 Department of Gerontological Evaluation, Center for Gerontology and Social Science, National Center for Geriatrics and Gerontology, Aichi, Japan; 8 Harvard School of Public Health, Department of Social and Behavioral Sciences, Boston, MA, USA; 9 The University of Tokyo, School of Public Health, Tokyo, Japan

**Keywords:** living arrangement, depressive symptoms, Japan, aged, social cohesion

## Abstract

**Background:**

There is little longitudinal evidence on the impact of specific living arrangements (ie, who individuals live with) on mental health among older adults, and no studies have examined the modifying effect of residential social cohesion level on this association. We aimed to examine the association between living arrangements and depressive symptoms and whether this association varies with residential neighborhood social cohesion level among 19,656 men and 22,513 women aged 65 years and older in Japan.

**Methods:**

We analyzed the association between baseline living arrangements in 2010 and depressive symptoms in 2013. We calculated gender-specific odds ratios (ORs) of living arrangements for depressive symptoms using a logistic regression and conducted subgroup analyses by neighborhood social cohesion level.

**Results:**

Among men (but not women), living alone (OR 1.43; 95% confidence intervals [CI], 1.18–1.73) and living with spouse and parent (OR 1.47, 95% CI, 1.09–1.98) were associated with increased odds of depressive symptoms compared with living with a spouse only. Living with spouse and child was a risk for men in the young age group but a protective factor for women. We also identified that the negative impact of living arrangements on depressive symptoms was attenuated in neighborhoods with higher levels of social cohesion.

**Conclusions:**

Living arrangements are associated with risk of depressive symptoms among men and women; these associations differ by gender and neighborhood social cohesion level. Our results suggest the need to pay more attention to whether individuals live alone, as well as who individuals live with, to prevent depressive symptoms among older adults.

## INTRODUCTION

Major depressive disorder is a primary cause of disability, as measured by years lived with disabilities.^1^ Depression in later life decreases individuals’ quality of life in terms of both psychological and physical health^2^ and increases the risk of premature death.^3^ In Japan, the number of older people with mood disorder and depression has substantially increased in recent years.^4^ Moreover, the population is rapidly aging, and it has been predicted that one in three Japanese people will be aged ≥65 years by 2030.^5^ Therefore, there needs to be a greater focus on mental health among older adults to reduce the individual and social burden of these diseases.

Previous studies have reported an association between living arrangement and mental health^6^^–^^12^ and agree that older adults living alone are at higher risk of experiencing deteriorations in mental health. Most studies conducted in Western countries on living arrangements among older adults have focused on whether individuals live alone or not. Studies in Asian countries (including Japan) have also examined detailed living arrangement (ie, who individuals live with) and depressive symptoms.^6^^,^^8^ However, to the best of our knowledge, there are few longitudinal studies on the association between variation in living arrangements and risk of developing depressive symptoms among older adults, and no such studies in Asia.

Living with someone has both advantages and disadvantages. Receiving various types of social support through cohabitants may positively impact their mental health,^6^^,^^13^ while relational conflicts and extra duties and responsibilities for cohabitants may negatively affect their mental health.^14^ In addition, impact of living arrangements could differ by gender, particularly in societies characterized by strong gender role norms (ie, the male bread-winner model).^15^ In such societies, women are generally more likely to adopt the role of providing a various types of social support for family members at home compared to men.^16^ Thus, we hypothesized that types of living arrangement affect people’s mental health differently, and the impact could differ by gender.

Social capital, defined as the resources that individuals access through their social networks, has been identified as a crucial social determinant of health.^17^ These social resources comprise trust between people in a network, the exchange of information, instrumental support, emotional support, and social reinforcement. Several studies have examined the effect of social capital on mental health among older adults,^14^^,^^18^^–^^20^ but few have investigated the interactive effect of social capital and other social factors. One study examined the interactive effect of marital status on the association between neighborhood disorder and depression among older adults and demonstrated that social relationships with marital partners buffer the association between social disorder and depression.^18^ In other words, residential social characteristics may affect the association between individual living conditions and mental health. Thus, we hypothesized that one aspect of social capital, social cohesion, could affect the association between living arrangements and depressive symptoms. For example, a high level of community social cohesion may mitigate loneliness, increase social support, or reduce the likelihood of social exclusion among individuals living alone or living without a spouse, which in turn may reduce the negative impact of living arrangements on mental health.

The objectives of this study were to investigate the associations of living arrangements (living alone; with spouse only; with spouse and parent(s); with spouse and child; with spouse, parent(s), and child; with parent(s) and/or child without spouse; or other arrangements) with depressive symptoms over a 3-year follow-up period among older Japanese adults. We aimed to answer the following specific research questions:

1) Does the risk of developing depressive symptoms differ according to living arrangements among Japanese men and women aged 65 years and older?
2) Is the association between depressive symptoms and living arrangements modified by gender?
3) Is the association between depressive symptoms and living arrangements modified by the level of neighborhood social cohesion?

## MATERIALS AND METHODS

### Study population

This study used longitudinal data from the Japan Gerontological Evaluation Study (JAGES) conducted in 2010 and 2013. Details of the study procedure have been described elsewhere.^22^ Briefly, the baseline sample in 2010 comprised 92,272 participants (response rate: 65%). Among them, 77,714 participants were targeted in the follow-up survey after the exclusion of participants who had died, received benefits from public long-term care insurance, or moved to another municipality during the follow-up period. Approximately 80% of the participants (*n* = 62,438) completed the follow-up self-report questionnaire in 2013.

Of these 62,438 men and women, we excluded the following: those who reported limitations in activities of daily living (defined as inability to walk, bathe, or use the toilet without assistance in 2010 or missing information on activities of daily living; *n* = 2,007), those with depressive symptoms (defined as a score of ≥5 on the Geriatric Depression Scale [GDS] at baseline; *n* = 15,125), those with missing information about depressed mood in 2010 and/or 2013 (*n* = 1,871), and those with missing information about living arrangements in 2010 (*n* = 1,149). We included the remaining 19,656 men and 22,513 women as our final study population.

The JAGES protocol was approved by the Ethics Committee on Research of Human Subjects at Nihon Fukushi University (No. 10-05). Use of the data for this study was approved by the Ethics Committee of the University of Tokyo, Faculty of Medicine (No. 10555).

### Primary predictor: living arrangements

Living arrangements were assessed using a self-reported baseline questionnaire. Participants responded to the question “Who do you live with” by choosing all the applicable options from the following: (a) living alone, (b) spouse, (c) child, (d) child-in-law, (e) grandchild, (f) parent(s), (g) parent(s)-in-law, (h) siblings, and (i) others. Based on the responses, we created seven types of living arrangement: (1) living with spouse only; (2) living alone; (3) living with spouse and parent(s); (4) living with spouse and child; (5) living with spouse, child, and parent(s); (6) living with parent(s) and/or child but not spouse; and (7) other living arrangements.

### Outcome: depressive symptoms

Participants were followed up to 2013. The endpoint of this study was depressive symptoms assessed with the Japanese short version of the GDS (the GDS-15)^23^ using a simple yes/no format suitable for self-administration.^21^ The GDS is a well-known instrument to measure depression among older adults and has been extensively validated and used for healthy older adults in community setting; the GDS score was found to have a sensitivity 88–92% and specificity of 62–81% compared with a structured clinical interview for depression.^23^ Following previous research,^22^^–^^25^ those with a score of ≥5 on the GDS in 2013 were considered to have newly developed depressive symptoms during the follow-up period.

### Modifying factor: neighborhood social cohesion level

For the subgroup analysis, we created a neighborhood social cohesion variable using a validated neighborhood social cohesion scale derived from Saito et al.^26^ Briefly, school district was defined as level of neighborhood and a measure of neighborhood social capital was generated by using factor analysis. The analysis produced three social capital components, one of which was social cohesion. Social cohesion was measured by summing up the scores on three questions about community trust, reciprocity, and community attachment for each school district, following our previous studies.^26^ The total number of school districts was 525 in this study. For the subgroup analysis, we created two social cohesion groups using the median: high and low. We did not calculate a social cohesion score for school districts with a small number of households (less than 25; *n* = 368) but treated these as missing data for this variable.

### Covariates

Age (years), GDS score at baseline, age group (60–74 years, 75 years and older), years of educational attainment (9 years or less, 10–12 years, 13 years or more), equivalent household income groups (0–1.99 million yen, 2–3.99 million yen, and 4 million yen and more per year), employment status (working, retired, or never worked), receiving treatment for any disease (yes/no), poor self-rated health (yes/no), time spent walking per day, and residential area (municipality; *n* = 24) at baseline were treated as confounding factors.

Social support exchange was hypothesized to be a mediating factor. Social support was assessed using the following questions: “Is there someone who listens to your concerns and complaints?” (Emotional support receipt), “Is there someone whose concerns and complaints you listen to?” (Emotional support provision), “Is there someone who helps and takes care of you when you are sick in bed?” (Instrumental support receipt), and “Is there someone who you help and take care of when s/he is sick in bed?” (Instrumental support provision). Responses to each question were classified as “Yes” or “No.”

### Statistical analysis

Proportions and mean values of GDS score, age, sociodemographic factors, and other covariates were calculated by gender as well as by living arrangements. We estimated gender-specific multivariable odds ratios (ORs) and 95% confidence intervals (CIs) for depressive symptoms according to living arrangements using men and women who lived with a spouse only as the reference group. We tested statistical interaction using cross-product terms for living arrangement and gender. Subgroup analysis by age (60–74 years group or 75 years and older group) was also performed. To examine if the identified associations were modified by the level of neighborhood social cohesion, we conducted subgroup analysis by neighborhood social cohesion level among those aged 65–74 years. We further included social support variables in the model in order to examine if social support could explain the impact of neighborhood social cohesion on the associations. Analyses were performed using SAS, version 9.4 (SAS Institute, Inc., Cary, NC, USA).

## RESULTS

During the mean follow-up period of 2.6 years, 2,577 men (13.0%) and 2,897 women (12.5%) developed depressive symptoms (Table 1). The proportions of women living alone and living with child and/or parent without spouse were higher than those of men. The proportion of men living with spouse and child was higher than that of women. The distributions of depressive symptoms differed by living arrangements. Moreover, the distribution of educational attainment level, household equivalent income level, working status, receiving treatment for any disease, poor self-rated health, time spent walking per day, social support exchange, mean age, and mean GDS score at baseline differed according to living arrangements among both men and women.

**Table 1.** Characteristics of subjects in the longitudinal samples of older Japanese men (*n* = 19,656) and women (*n* = 22,513) according to living arrangement

	MEN (*n* = 19,656)		Living arrangement														*P*-value for difference of living arrangement

			With spouse only (*n* = 9,468, 48%)		Living alone (*n* = 983, 5%)		With spouse and parent(s) (*n* = 447, 2%)		With spouse and child (*n* = 6383, 32%)		With spouse, child and parent(s) (*n* = 403, 2%)		Child only, parent(s) only, or child and parent(s) only (*n* = 1,736, 9%)		Others (*n* = 218, 1%)		
							
	*n*	%	*n*	%	*n*	%	*n*	%	*n*	%	*n*	%	*n*	%	*n*	%	
**MEN**																	
**Depressive symptoms (GDS score ≥5) (2013)**																	<.0001
Yes	2,490	13	1,144	12	174	18	60	13	797	12	31	8	253	15	31	14	
**Younger age group**																	<.0001
65–74 years old	13,090	67	6,341	67	566	58	412	92	4,330	68	373	93	933	54	135	62	
**Years of education attainment**																	<.0001
13 years and more	5,121	26	2,764	29	250	25	165	37	1,507	24	121	30	284	16	30	14	
10–12 years	6,849	35	3,469	37	310	32	168	38	2,220	35	147	36	480	28	55	25	
9 years and less	7,447	38	3,140	33	402	41	109	24	2,597	35	129	32	945	54	125	57	
missing	239	1	113	1	21	2	5	1	59	1	6	2	27	2	8	4	
**Household equivalent income**																	<.0001
4 million yen and higher	2,510	13	866	9	101	10	56	13	1,155	18	84	21	233	13	15	7	
2–3.99 million yen	8,303	42	4,004	42	414	42	235	53	2,847	45	169	42	577	33	57	26	
1.99 million yen and lower	7,221	37	4,099	43	302	31	136	30	1,795	28	124	31	658	38	107	49	
missing	1,622	8	517	6	166	17	20	4	586	9	26	6	268	15	39	18	
**Working status**																	<.0001
Working	6,070	31	2,738	29	238	24	178	40	2,172	34	186	46	504	29	54	25	
Retired	11,872	60	6,016	63	599	61	246	55	3,694	58	195	48	982	57	138	63	
Never work	592	3	283	3	58	6	6	1	152	2	5	1	79	5	9	4	
missing	1,122	6	449	5	88	6	17	4	365	6	17	4	169	9	17	8	
**Disease treatment**						9											<.0001
No	5,301	27	2,452	26	284	29	157	35	11,756	28	121	30	467	27	64	29	
Yes	13,050	66	6,389	67	615	63	273	61	4,236	66	265	66	1,140	66	132	61	
Missing	1,305	7	645	7	84	9	17	4	391	6	17	4	129	7	22	10	
**Poor self-rated health**																	0.05
No	17,535	89	8,442	89	890	90	420	94	5,689	89	362	90	1,536	88	196	90	
Yes	1,992	10	990	10	87	9	27	6	642	10	38	9	186	11	22	10	
Missing	129	1	54	1	6	1	0	0	52	1	3	1	14	1	0	0	
**Walking time period per day**																	<.0001
29 min and less	4,642	24	2,195	23	251	26	85	19	1,473	23	96	24	471	27	71	33	
30–59 min	6,806	35	3,469	37	328	33	152	34	2,119	33	113	28	550	32	75	34	
60–89 min	3,597	18	1,747	18	189	19	87	19	1,174	18	84	21	286	17	30	14	
90 min and more	3,790	19	1,670	18	167	17	107	24	1,383	22	92	23	335	19	36	17	
missing	821	4	405	4	48	5	16	4	234	4	18	4	94	5	6	3	
**Emotional social support receipt**																	<.0001
No	1,045	5	410	4	183	19	13	3	255	4	9	2	145	8	30	14	
Yes	17,645	90	8,627	91	727	74	417	93	5,852	92	377	94	1,466	85	179	82	
Missing	966	5	449	5	73	7	17	4	276	4	17	4	125	7	9	4	
**Emotional social support provision**																	<.0001
No	975	5	359	4	171	17	13	3	242	4	11	3	150	9	29	13	
Yes	17,735	90	8,686	92	748	76	417	93	5,873	92	376	93	1,461	84	174	80	
Missing	946	5	441	5	64	7	17	4	268	4	16	4	125	7	15	7	
**Instrumental social support receipt**																	<.0001
No	488	2	85	1	280	28	2	1	39	1	2	1	65	4	15	7	
Yes	18,379	94	9,045	95	643	65	430	96	6,115	96	386	96	1,568	90	192	88	
Missing	789	4	356	4	60	7	15	3	229	4	15	4	103	6	11	5	
**Instrumental social support provision**																	<.0001
No	1,372	7	346	4	422	43	15	3	272	4	7	2	264	15	46	21	
Yes	17,271	88	8,710	92	482	49	412	92	5,811	91	378	94	1,319	76	159	73	
Missing	1,013	5	430	5	79	8	20	5	300	5	18	5	153	9	13	6	

	**Mean**	**SD**	**Mean**	**SD**	**Mean**	**SD**	**Mean**	**SD**	**Mean**	**SD**	**Mean**	**SD**	**Mean**	**SD**	**Mean**	**SD**	
**Age**	72.6	5.5	72.6	5.2	73.8	6.1	69.0	3.6	72.4	5.5	68.7	3.6	74.5	6.3	73.6	5.8	<.0001
**GDS score in 2010**	1.6	1.3	1.6	1.3	1.8	1.4	1.5	1.3	1.6	1.3	1.5	1.3	1.7	1.3	1.9	1.4	0.0002



	WOMEN (*n* = 22,513)		Living arrangement														*P*-value for difference of living arrangement

			With spouse only (*n* = 7,805, 35%)		Living alone (*n* = 3,355, 15%)		With spouse and parent(s) (*n* = 227, 1%)		With spouse and child (*n* = 5232, 23%)		With spouse, child and parent(s) (*n* = 177, 1%)		Child only, parent(s) only, or child and parent(s) only (*n* = 5294, 24%)		Others (*n* = 423, 2%)		
							
	*n*	%	*n*	%	*n*	%	*n*	%	*n*	%	*n*	%	*n*	%	*n*	%	
**WOMEN**																	
**Depressive symptoms (GDS score ≥5) (2013)**																	<.0001
Yes	2,767	12	915	12	471	14	21	9	548	11	19	11	726	14	67	16	
**Younger age group**																	<.0001
65–74 years old	14,833	66	5,963	77	1,788	53	216	95	3,915	75	163	92	2,544	48	224	53	
**Years of education attainment**																	<.0001
13 years and more	3,236	14	1,346	17	556	17	51	23	661	13	27	15	521	10	74	17	
10–12 years	8,505	38	3,289	42	1,288	38	111	49	1,934	37	75	42	1,652	31	156	37	
9 years and less	10,340	46	3,060	39	1,418	42	61	27	2,566	49	75	42	2,983	57	177	42	
missing	432	2	110	1	93	3	4	2	71	1	0	0	138	3	16	4	
**Household equivalent income**																	<.0001
4 million yen and higher	2,366	11	582	8	146	4	21	9	852	16	31	18	716	14	18	4	
2–3.99 million yen	7,607	34	3,046	39	869	26	124	55	1,935	37	61	34	1,480	28	92	22	
1.99 million yen and lower	8,475	38	3,451	44	1,515	45	64	28	1,442	28	56	32	1,735	33	212	50	
missing	4,065	18	726	9	825	25	18	8	1,003	19	29	16	1,363	26	101	24	
**Working status**																	<.0001
Working	3,888	17	1,286	16	558	17	54	24	1,057	20	56	32	816	15	61	14	
Retired	11,550	51	4,307	55	1,753	52	120	53	2,633	50	76	43	2,420	46	241	57	
Never work	3,735	17	1,287	16	525	16	34	15	855	16	24	14	964	18	46	11	
missing	3,340	15	925	12	519	15	19	8	687	13	21	12	1,094	21	75	18	
**Disease treatment**																	<.0001
No	5,533	25	2,147	28	755	23	63	28	1,372	26	49	28	1,034	20	113	27	
Yes	15,168	67	5,076	65	2,278	68	153	67	3,515	67	120	68	3,772	71	257	61	
Missing	1,812	8	582	7	322	10	11	5	348	7	8	5	488	9	53	13	
**Poor self-rated health**																	0.27
No	20,259	90	7,014	90	3,065	91	203	89	4,716	90	159	90	1,726	89	376	89	
Yes	1,997	9	706	9	251	8	22	10	461	9	16	9	502	10	39	9	
Missing	257	1	85	1	39	1	2	1	55	1	2	1	66	1	8	2	
**Walking time period per day**																	<.0001
29 min and less	6,027	27	1,921	25	917	27	47	21	1,350	26	44	25	1,630	31	118	28	
30–59 min	7,886	35	2,894	37	1,255	37	87	38	1,724	33	46	26	1,750	33	130	31	
60–89 min	3,534	16	1,287	16	535	16	33	15	801	15	27	15	788	15	63	15	
90 min and more	3,754	17	1,285	16	454	13	46	20	1,055	20	53	30	786	15	75	18	
missing	1,312	6	418	5	194	6	14	6	302	6	7	4	340	6	37	9	
**Emotional social support receipt**																	<.0001
No	429	2	96	1	148	4	1	1	48	1	0	0	120	2	16	4	
Yes	21,061	94	7,400	95	3,038	91	213	94	4,987	95	173	98	4,882	92	368	87	
Missing	1,023	5	309	4	169	5	13	6	197	4	4	2	292	6	39	9	
**Emotional social support provision**																	<.0001
No	692	3	165	2	157	5	2	1	81	2	2	1	264	5	21	5	
Yes	20,619	92	7,281	93	3,023	90	211	93	4,906	94	171	97	4,669	88	358	85	
Missing	1,202	5	359	5	175	5	14	6	245	5	4	2	361	7	44	10	
**Instrumental social support receipt**																	<.0001
No	658	3	112	1	400	12	1	1	39	1	1	1	80	2	25	6	
Yes	20,866	93	7,401	95	2,762	82	213	94	4,981	95	172	97	4,982	94	355	84	
Missing	989	4	292	4	193	6	13	6	212	4	4	2	232	4	43	10	
**Instrumental social support provision**																	<.0001
No	1,828	8	177	2	762	23	0	0	120	2	2	1	721	14	46	11	
Yes	19,135	85	7,285	93	2,267	68	214	94	4,857	93	168	95	4,024	76	320	76	
Missing	1,550	7	343	4	326	10	13	6	255	5	7	4	549	10	57	13	

	**Mean**	**SD**	**Mean**	**SD**	**Mean**	**SD**	**Mean**	**SD**	**Mean**	**SD**	**Mean**	**SD**	**Mean**	**SD**	**Mean**	**SD**	
**Age**	72.7	5.5	71.3	4.5	74.4	5.6	68.5	3.0	71.5	4.8	68.6	3.5	75.3	6.2	74.4	5.9	<.0001
**GDS score in 2010**	1.7	1.3	1.7	1.3	1.8	1.3	1.7	1.3	1.7	1.3	1.6	1.4	1.8	1.3	1.9	1.3	<.0001

Table 2 shows the gender-specific multivariable ORs of depressive symptoms according to living arrangements with living with spouse only as a reference. Among men, living alone (OR 1.43; 95% CI, 1.18–1.73) and living with spouse and parent(s) (OR 1.47; 95% CI, 1.09–1.98) were associated with increased odds of developing depressive symptoms; however, no such associations were identified among women (the *P*-values for the gender interaction were 0.07 and 0.09, respectively). Living with spouse and child had a protective effect for women (OR 0.84; 95% CI, 0.74–0.95) but not for men (OR 1.08; 95% CI, 0.97–1.20) (the *P*-value for the gender interaction was 0.18). Compared with women living with spouse and child, women living alone showed increased odds of having depressive symptoms (OR 1.19; 95% CI, 1.06–1.35; not shown in the table).

**Table 2.** Gender-specific adjusted odds ratios of living arrangement for depressive symptoms

	ALL *n* = 42,169								*P*-value for interaction of gender

	Men (*n* = 19,656)				Women (*n* = 22,513)				
	
	*n*	*n* of case	OR^a^	95% CI	*n*	*n* of case	OR^a^	95% CI	
**Living arrangement**									
With spouse only	9,468	1,144	1.00		7,805	915	1.00		
Living alone	983	174	1.43	(1.18, 1.73)	3,355	471	1.04	(0.91, 1.18)	0.07
With spouse and parent(s)	447	60	1.47	(1.09, 1.98)	227	21	0.82	(0.51, 1.32)	0.09
With spouse and child	6,383	797	1.08	(0.97, 1.20)	5,232	548	0.84	(0.74, 0.95)	0.18
With spouse, child and parent(s)	403	31	0.72	(0.49, 1.06)	177	19	0.95	(0.57, 1.58)	0.12
Child only, parent(s) only, or child and parent(s) only	1,736	253	1.12	(0.96, 1.32)	5,294	726	0.95	(0.84, 1.07)	0.62
Others	218	31	0.97	(0.65, 1.46)	423	67	1.11	(0.83, 1.48)	0.27
									
**GDS score in 2010**	19,656	2,490	1.84	(1.78, 1.91)	22,513	2,767	1.91	(1.85, 1.98)	
**Age**	19,656	2,490	1.02	(1.00, 1.03)	22,513	2,767	1.02	(1.01, 1.04)	
**Age group**									
65–74 years old	13,090	1,475	1.00		14,833	1,675	1.00		
75 years and older	6,566	1,015	1.04	(0.88, 1.23)	7,680	1,092	0.90	(0.77, 1.06)	
**Years of education attainment**									
13 years and more	5,121	458	1.00		3,236	320	1.00		
10–12 years	6,849	851	1.30	(1.14, 1.47)	8,505	903	0.99	(0.86, 1.14)	
9 years and less	7,447	1,140	1.37	(1.20, 1.56)	10,340	1,469	1.19	(1.03, 1.36)	
missing	239	41	1.62	(1.11, 2.36)	432	75	1.34	(0.99, 1.81)	
**Household equivalent income**									
4 million yen and higher	2,510	194	1.00		2,366	195	1.00		
2–3.99 million yen	8,303	881	1.17	(0.98, 1.39)	7,607	767	1.14	(0.96, 1.35)	
1.99 million yen and lower	7,221	1,154	1.54	(1.29, 1.83)	8,475	1,194	1.37	(1.15, 1.62)	
missing	1,622	261	1.43	(1.16, 1.77)	4,065	611	1.39	(1.16, 1.67)	
**Working status**									
Working	6,070	655	1.00		3,888	441	1.00		
Retired	11,872	1,562	0.99	(0.89, 1.10)	11,550	1,358	0.86	(0.76, 0.97)	
Never work	592	116	1.21	(0.95, 1.53)	3,735	485	0.89	(0.76, 1.03)	
missing	1,122	157	0.85	(0.69, 1.04)	3,340	483	0.91	(0.78, 1.06)	
**Disease treatment**									
No	5,301	526	1.00		5,533	525	1.00		
Yes	13,050	1,800	1.20	(1.07, 1.34)	15,168	1,994	1.12	(1.01, 1.25)	
Missing	1,305	164	1.04	(0.86, 1.28)	1,812	248	1.10	(0.92, 1.31)	
**Poor self-rated health**									
No	17,535	1,963	1.00		20,259	2,266	1.00		
Yes	1,992	501	1.70	(1.51, 1.93)	1,997	466	1.57	(1.39, 1.78)	
Missing	129	26	1.46	(0.92, 2.32)	257	35	0.97	(0.66, 1.42)	
**Walking time period per day**									
29 min and less	4,642	732	1.00		6,027	915	1.00		
30–59 min	6,806	822	0.93	(0.83, 1.04)	7,886	947	0.93	(0.83, 1.03)	
60–89 min	3,597	414	0.96	(0.84, 1.10)	3,534	375	0.86	(0.75, 0.98)	
90 min and more	3,790	406	0.94	(0.82, 1.08)	3,754	370	0.85	(0.74, 0.98)	
missing	821	116	1.05	(0.83, 1.31)	1,312	160	0.79	(0.65, 0.96)	

CI, confidence interval; GDS, Geriatric Depression Scale; OR, odds ratio.

^a^Adjusted by all variables in the table. Residential area was also adjusted using a fixed model (ie, using 23 dummy variables).

We identified associations between living arrangements and depressive symptoms among both men and women in the younger age group, but found no statistically significant associations in the older age group (Table 3). In particular, men living with spouse and child was a significant risk of depressive symptoms for men aged 65–74 years. Thus, we decided to use only the younger age group (65–74 years old) for further subgroup analysis.

**Table 3.** Gender-specific adjusted odds ratios of living arrangement for depressive symptoms

	ALL *n* = 42,169									

	Men (*n* = 19,656)				*P*-value for interaction of age group	Women (*n* = 22,513)				*P*-value for interaction of age group
	
	*n*	*n* of case	OR^a^	95% CI		*n*	*n* of case	OR^a^	95% CI	
**Living arrangement**										
**Age 65–74 years**										
With spouse only	6,341	663	1.00			5,983	663	1.00		
Living alone	566	107	1.79	(1.40, 2.29)	0.03	1,788	253	1.16	(0.98, 1.37)	0.95
With spouse and parent(s)	412	56	1.68	(1.23, 2.30)	0.23	216	20	0.86	(0.53, 1.41)	0.96
With spouse and child	4,330	496	1.19	(1.04, 1.36)	0.53	3,915	375	0.81	(0.71, 0.94)	0.93
With spouse, child and parent(s)	373	25	0.68	(0.44, 1.05)	0.03	163	19	1.08	(0.64, 1.80)	0.95
Child only, parent(s) only, or child and parent(s) only	933	110	1.08	(0.86, 1.35)	0.38	2,544	304	0.96	(0.82, 1.13)	0.94
Others	135	18	1.05	(0.62, 1.79)	0.71	224	41	1.48	(1.02, 2.16)	0.96

**Age ≥75 years**										
With spouse only	3,145	481	1.00			1,822	252	1.00		
Living alone	417	67	1.03	(0.76, 1.40)		1,567	218	0.88	(0.71, 1.09)	
With spouse and parent(s)	35	4	0.82	(0.27, 2.49)		11	1	0.43	(0.05, 3.73)	
With spouse and child	2,053	301	0.91	(0.77, 1.09)		1,317	173	0.88	(0.70, 1.10)	
With spouse, child and parent(s)	30	6	1.62	(0.62, 4.26)		14	0	NA		
Child only, parent(s) only, or child and parent(s) only	83	143	1.14	(0.91, 1.44)		2,750	422	0.87	(0.72, 1.06)	
Others	88	13	0.88	(0.46, 1.66)		199	26	0.74	(0.46, 1.17)	

CI, confidence interval; OR, odds ratio.

^a^Adjusted by GDS score in 2010, age, age group, years of education attainment, household income, working status, disease treatment, poor self-rated health, and walking time period per day. Residential area was also adjusted using a fixed model (ie, using 23 dummy variables).

Table 4 shows the gender-specific ORs for depressive symptoms according to living arrangements by neighborhood social cohesion level in men and women in the younger age group (65–74 years old). The negative impact of living arrangements on depressive symptoms was attenuated in neighborhoods with higher levels of social cohesion among men and women aged 65–74 years, although the multiplicative interaction was not significant (*P*-value for the interaction of social cohesion level = 0.66). The ORs of living alone for men were 2.01 (95% CI, 1.44–2.82) in the less socially cohesive neighborhood group and 1.46 (95% CI, 0.98–2.18) in the more socially cohesive neighborhood group. In addition, the OR of living alone for men in the less socially cohesive neighborhood group was significantly reduced by adjusting for social support variables (OR 1.54; 95% CI, 1.04–2.30).

**Table 4.** Gender-specific adjusted odds ratios of living arrangement for depressive symptoms by social cohesion level among men and women aged 65–74 years

Living arrangement	*n*	*n* of case	Model 1		Model 2		*P*-value for interaction of social cohesion level
	
			OR	95% CI	OR	95% CI	
**Men (*n* = 12,572)**							
**Social cohesion level**							
**LOW**							
With spouse only	3,330	334	1.00		1.00		
Living alone	289	60	2.01	(1.44, 2.82)	1.54	(1.04, 2.30)	0.66
With spouse and parent(s)	162	21	1.80	(1.09, 2.97)	1.82	(1.10, 3.00)	0.57
With spouse and child	1,979	238	1.29	(1.07, 1.56)	1.29	(1.07, 1.57)	0.52
With spouse, child and parent(s)	135	11	0.80	(0.41, 1.55)	0.81	(0.42, 1.57)	0.83
Child only, parent(s) only, or child and parent(s) only	410	51	1.11	(0.80, 1.56)	1.05	(0.75, 1.48)	0.33
Others	77	13	1.54	(0.81, 2.93)	1.39	(0.72, 2.67)	0.28
**HIGH**							
With spouse only	2,769	300	1.00		1.00		
Living alone	235	36	1.46	(0.98, 2.18)	1.27	(0.81, 2.01)	
With spouse and parent(s)	236	32	1.54	(1.01, 2.34)	1.54	(1.01, 2.36)	
With spouse and child	2,192	242	1.10	(0.91, 1.34)	1.10	(0.90, 1.34)	
With spouse, child and parent(s)	227	13	0.56	(0.31, 1.01)	0.55	(0.30, 1.00)	
Child only, parent(s) only, or child and parent(s) only	481	57	1.08	(0.78, 1.49)	1.05	(0.76, 1.45)	
Others	50	5	0.70	(0.26, 1.88)	0.62	(0.23, 1.68)	

**Women (*n* = 14,266)**							
**Social cohesion level**							
**LOW**							
With spouse only	3,078	356	1.00		1.00		
Living alone	955	140	1.19	(0.95, 1.50)	1.08	(0.85, 1.38)	0.62
With spouse and parent(s)	90	9	0.81	(0.39, 1.68)	0.81	(0.39,1.68)	0.82
With spouse and child	1,712	163	0.77	(0.62, 0.95)	0.77	(0.62, 0.95)	0.87
With spouse, child and parent(s)	64	10	1.45	(0.70, 2.98)	1.43	(0.69, 2.96)	0.23
Child only, parent(s) only, or child and parent(s) only	1,183	128	0.80	(0.64, 1.01)	0.78	(0.62, 0.99)	0.13
Others	126	20	1.19	(0.71, 2.01)	1.11	(0.65, 1.89)	0.31
**HIGH**							
With spouse only	2,656	287	1.00		1.00		
Living alone	737	101	1.11	(0.85, 1.45)	1.02	(0.78, 1.35)	
With spouse and parent(s)	117	11	0.91	(0.47, 1.79)	0.91	(0.46, 1.78)	
With spouse and child	2,075	188	0.80	(0.64, 0.98)	0.80	(0.64, 0.98)	
With spouse, child and parent(s)	95	9	0.84	(0.40, 1.77)	0.86	(0.41, 1.81)	
Child only, parent(s) only, or child and parent(s) only	1,290	163	1.07	(0.86, 1.35)	1.07	(0.85, 1.34)	
Others	88	18	1.70	(0.95, 3.02)	1.60	(0.90, 2.86)	

CI, confidence interval; GDS, Geriatric Depression Scale; OR, odds ratio.

Model 1: adjusted by GDS score in 2010, age, years of education attainment, household income, working status, disease treatment, poor self-rated health, and walking time period per day. Residential area was also adjusted using a fixed model (ie, using 23 dummy variables).

Model 2: Model 1+ emotional support receipt, emotional support provision, instrumental support receipt, and instrumental support provision.

## DISCUSSION

In this study of Japanese older adults, living arrangements were significantly associated with risk of depressive symptoms. Our results indicated that the association between living arrangements and depressive symptoms differs by gender. We found that men living alone and living with a spouse and parent(s) had higher odds of developing depressive symptoms than those living with their spouse only; however, no such association was identified among women. In contrast, women living with a spouse and child had lowered odds of developing depressive symptoms compared with those living with a spouse only, whereas increased odds were identified among men in the younger age group. Moreover, our results suggest that neighborhood social cohesion level may affect the associations between living arrangements and depressive symptoms. The increased odds of depressive symptoms for those living alone were slightly attenuated in those living in neighborhoods with greater social cohesion.

There is good evidence that living alone is a risk factor for depressive symptoms among older adults.^7^^,^^27^ However, to the best of our knowledge, there are few longitudinal studies on specific living arrangements and depressive symptoms among older adults. A cross-sectional study in South Korea reported that older men and women living with spouse only were the least likely to have depressive symptoms; however, living with other family members in addition to a spouse, as well as living alone, were associated with higher odds of depressive symptoms among men and women.^8^ Another cross-sectional study in Japan also indicated that living with family members other than a spouse was associated with increased odds of psychological distress among men and women aged 65–74 years.^6^

Our results are partly consistent with these previous results; living alone was significant risk of developing depressive symptoms. However, while previous studies showed no gender differences in the association between living arrangement and depressive symptoms, we identified clear gender difference in the effect of living with spouse and living with child/parent. Living with a spouse only was beneficial for mental health among men, but it was not necessarily true for women. For women, living with a spouse and child was most beneficial factor, but it seemed to be a risk for men at least in the younger age group. In addition, living with a spouse and parent(s) was risk for depressive symptoms for men but not for women. Our results suggested that who an individual lives with, not just whether they live with someone, is important for mental health among older adults, and suggested significant gender differences in the association between living arrangements and depressive symptoms among older Japanese adults.

The gender differences identified may be a result of differences in the expected social roles of men and women in Japanese society, which is characterized by strong gender role norms.^15^ Under such gendered norms, men may feel role conflicts when they cannot fulfill their role responsibility, such as provision of financial support for family members, when they retire. In contrast, because women are expected to take care of their family members, this may shape their identity within the family; living with their child/parent(s) may enhance women’s roles.^16^ Moreover, women are generally more likely to adopt the role of providing a range of social support to their spouse under such social norms. Therefore, for men, living with a spouse may mean that they have someone to take care of them; for women, living with a spouse may mean that they have someone who needs their care. These different roles may be the basis of the identified gender differences in the associations between living arrangements and depressive symptoms.

Another explanation for these findings may be differences in how men and women construct and maintain social networks. Older adults are likely to be vulnerable to social isolation because they are more likely to lose their social ties.^28^ However, women living alone are not necessarily socially isolated and often show better psychological health compared with those living with a spouse.^29^ Constructing social relationships is beneficial for mental health among older adults.^30^ Women are likely to maintain their active social networks with their friends, immediate family, and other relatives and experience more social support regardless of their marital status,^30^ whereas older men tend to mainly have relationships with their spouses.^28^

We found that living in socially cohesive neighborhoods may prevent the occurrence of depressive symptoms among people living alone. One possible explanation for this is that cohesive communities may provide more social support for residents,^17^ which may reduce the likelihood of social isolation and social exclusion among community members. As social isolation and social exclusion are risks for depressive symptoms,^31^ community cohesiveness may reduce the risk of depressive symptoms among people living alone. Indeed, the results of our mediating analysis indicated that the increased odds of depressive symptoms in individuals living alone in less socially cohesive neighborhoods could be explained by less social support exchange among people in those areas. Our results indicate that interventions to improve aspects of social cohesion may help to prevent depressive symptoms among older individuals.

This is one of the few longitudinal investigations to examine the association between living arrangements and risk of depressive symptoms. However, several limitations should be mentioned. First, we did not account for changes in our primary predictor or in other variables during the follow-up period. Second, this was an observational study and selection bias could not be ruled out. Unfortunately, we have no demographic information on those who did not participate in this cohort study, so no information on the direction of this selection bias was available. Third, residual confounding could have occurred from unmeasured confounding variables, such as family history of mental health. Fourth, measurement errors could also occur. Measurement error of our outcome was assumed to be non-differential and might have reduced the reliability of our result. Fifth, although it was a strength of our study design to have data on depressive symptoms at baseline and follow-up, our study population was limited to those who responded to both questionnaires, which may introduce some selection bias. Those who did not response to the following survey was likely to be older, have lower socioeconomic conditions, and poorer self-rated health, and to live with parent(s) and/or child but not spouse, compared to our study population, which did not indicate clear direction of this bias.

Although these cautions are necessary to interpret, our results suggest that public health practitioners and policy makers should pay more attention to whether individuals live alone, as well as who individuals live with. It also support that interventions to strengthen community social cohesion may be effective to prevent depressive symptoms of older community residents, regardless of their living arrangements. Given the increasing diversity in family conception, it is unrealistic to promote specific cohabitation statuses among older adults. Alternatively, community interventions to strengthen social cohesion may work; for example, by creating more opportunities of social informal gathering, such as “community salons”.^32^

## References

[1] Global Burden of Disease Study 2013 Collaborators Global, regional, and national incidence, prevalence, and years lived with disability for 301 acute and chronic diseases and injuries in 188 countries, 1990–2013: a systematic analysis for the Global Burden of Disease Study 2013. Lancet. 2015;386(9995):743–800. 10.1016/S0140-6736(15)60692-4 26063472 PMC4561509

[2] Beekman AT, Penninx BW, Deeg DJ, The impact of depression on the well-being, disability and use of services in older adults: a longitudinal perspective. Acta Psychiatr Scand. 2002;105(1):20–27. 10.1034/j.1600-0447.2002.10078.x 12086221

[3] Fiske A, Wetherell JL, Gatz M Depression in older adults. Annu Rev Clin Psychol. 2009;5:363–389. 10.1146/annurev.clinpsy.032408.153621 19327033 PMC2852580

[4] Japanese Ministry of Health LaW. Patient Survey 2014. Secondary Patient Survey 2014. (online) Available at: http://www.mhlw.go.jp/english/database/db-hss/ps.html. Accessed Nov 14, 2016.

[5] Muramatsu N, Akiyama H Japan: super-aging society preparing for the future. Gerontologist. 2011;51(4):425–432. 10.1093/geront/gnr067 21804114

[6] Kikuchi H, Takamiya T, Odagiri Y, Gender differences in association between psychological distress and detailed living arrangements among Japanese older adults, aged 65–74 years. Soc Psychiatry Psychiatr Epidemiol. 2014;49(5):823–830. 10.1007/s00127-013-0778-8 24126557

[7] Russell D, Taylor J Living alone and depressive symptoms: the influence of gender, physical disability, and social support among Hispanic and non-Hispanic older adults. J Gerontol B Psychol Sci Soc Sci. 2009;64(1):95–104. 10.1093/geronb/gbn002 19176487 PMC2654980

[8] Oh DH, Park JH, Lee HY, Association between living arrangements and depressive symptoms among older women and men in South Korea. Soc Psychiatry Psychiatr Epidemiol. 2015;50(1):133–141. 10.1007/s00127-014-0904-2 24912401

[9] Zunzunegui MV, Béland F, Otero A Support from children, living arrangements, self-rated health and depressive symptoms of older people in Spain. Int J Epidemiol. 2001;30(5):1090–1099. 10.1093/ije/30.5.1090 11689528

[10] Harris T, Cook DG, Victor C, Onset and persistence of depression in older people—results from a 2-year community follow-up study. Age Ageing. 2006;35(1):25–32. 10.1093/ageing/afi216 16303774

[11] Forsell Y Predictors for Depression, Anxiety and psychotic symptoms in a very elderly population: data from a 3-year follow-up study. Soc Psychiatry Psychiatr Epidemiol. 2000;35(6):259–263. 10.1007/s001270050237 10939425

[12] Joutsenniemi K, Martelin T, Martikainen P, Living arrangements and mental health in Finland. J Epidemiol Community Health. 2006;60(6):468–475. 10.1136/jech.2005.040741 16698975 PMC2563935

[13] Umberson D Gender, marital status and the social control of health behavior. Soc Sci Med. 1992;34(8):907–917. 10.1016/0277-9536(92)90259-S 1604380

[14] Bierman A Marital status as contingency for the effects of neighborhood disorder on older adults’ mental health. J Gerontol B Psychol Sci Soc Sci. 2009;64(3):425–434. 10.1093/geronb/gbp010 19251881 PMC2905133

[15] Shirahase S. *Social Inequality in Japan*. New York: Routledge; 2014.

[16] Reid J, Hardy M Multiple roles and well-being among midlife women: testing role strain and role enhancement theories. J Gerontol B Psychol Sci Soc Sci. 1999;54(6):S329–S338. 10.1093/geronb/54B.6.S329 10625968

[17] Kawachi I, Subramanian SV, Kim D. Social capital and health: a decade of progress and beyond. In: Kawachi I, Subramanian SV, Kim D, eds. *Social capital and Health*. New York: Springer; 2008:1–28.

[18] Kubzansky LD, Subramanian SV, Kawachi I, Neighborhood contextual influences on depressive symptoms in the elderly. Am J Epidemiol. 2005;162(3):253–260. 10.1093/aje/kwi185 15987730

[19] Beard JR, Cerdá M, Blaney S, Neighborhood characteristics and change in depressive symptoms among older residents of New York City. Am J Public Health. 2009;99(7):1308–1314. 10.2105/AJPH.2007.125104 19008519 PMC2696669

[20] Takagi D, Kondo K, Kondo N, Social disorganization/social fragmentation and risk of depression among older people in Japan: multilevel investigation of indices of social distance. Soc Sci Med. 2013;83:81–89. 10.1016/j.socscimed.2013.01.001 23333256

[21] Murayama H, Nofuji Y, Matsuo E, Are neighborhood bonding and bridging social capital protective against depressive mood in old age? A multilevel analysis in Japan. Soc Sci Med. 2015;124:171–179. 10.1016/j.socscimed.2014.11.042 25461874

[22] Tani Y, Fujiwara T, Kondo N, Childhood socioeconomic status and onset of depression among Japanese older adults: The JAGES Prospective Cohort Study. Am J Geriatr Psychiatry. 2016;24(9):717–726. 10.1016/j.jagp.2016.06.001 27569265

[23] Wada T, Ishine M, Kita T, Depression screening of elderly community-dwelling Japanese. J Am Geriatr Soc. 2003;51(9):1328–1329. 10.1046/j.1532-5415.2003.514207.x 12919256

[24] Burke WJ, Roccaforte WH, Wengel SP The short form of the Geriatric Depression Scale: a comparison with the 30-item form. J Geriatr Psychiatry Neurol. 1991;4(3):173–178. 1953971 10.1177/089198879100400310

[25] Murata C, Kondo K, Hirai H, Association between depression and socio-economic status among community dwelling elderly in Japan: the Aichi Gerontlogical Evaluation Study (JAGES). Health Place. 2008;14:406–414. 10.1016/j.healthplace.2007.08.007 17913562

[26] Saito M, Kondo N, Aida J, Development of an instrument for community-level health related social capital among Japanese older people: The JAGES Project. J Epidemiol. 2017;27(5):221–227. 10.1016/j.je.2016.06.005 28169107 PMC5394224

[27] Stone J, Evandrou M, Falkingham J The transition to living alone and psychological distress in later life. Age Ageing. 2013;42(3):366–372. 10.1093/ageing/aft006 23470713 PMC3633366

[28] Klinenberg E Social isolation, loneliness, and living alone: identifying the risks for public health. Am J Public Health. 2016;106(5):786–787. 10.2105/AJPH.2016.303166 27049414 PMC4985072

[29] Michael YL, Berkman LF, Colditz GA, Living arrangements, social integration, and change in functional health status. Am J Epidemiol. 2001;153(2):123–131. 10.1093/aje/153.2.123 11159156

[30] Koizumi Y, Awata S, Kuriyama S, Association between social support and depression status in the elderly: results of a 1-year community-based prospective cohort study in Japan. Psychiatry Clin Neurosci. 2005;59(5):563–569. 10.1111/j.1440-1819.2005.01415.x 16194259

[31] Cornwell EY, Waite LJ Social disconnectedness, perceived isolation, and health among older adults. J Health Soc Behav. 2009;50(1):31–48. 10.1177/002214650905000103 19413133 PMC2756979

[32] Hikichi H, Kondo N, Kondo K, Effect of a community intervention programme promoting social interactions on functional disability prevention for older adults: propensity score matching and instrumental variable analyses, JAGES Taketoyo study. J Epidemiol Community Health. 2015;69(9):905–910. 10.1136/jech-2014-205345 25888596 PMC4552922

